# Factors that promote and threaten Hope in caregivers of children with chronic conditions *

**DOI:** 10.1590/1518-8345.6366.3897

**Published:** 2023-05-12

**Authors:** Nayara Luiza Henriques, Juliana Barony da Silva, Zaida Borges Charepe, Patrícia Pinto Braga, Elysângela Dittz Duarte

**Affiliations:** 1 Universidade de Minas Gerais, Escola de Enfermagem. Belo Horizonte, MG, Brasil; 2 Universidade Federal de Minas Gerais, Grupo de Estudos sobre o Recém-nascido, Criança, Adolescentes e suas Famílias (RECRIA). Belo Horizonte, MG, Brasil; 3 Universidade Católica Portuguesa, Instituto de Ciências da Saúde. Lisboa; 4 Universidade Federal de São João del-Rei, Faculdade de Enfermagem. Belo Horizonte, MG, Brasil; 5 Universidade Federal de São João del-Rei, Núcleo de Estudos sobre Criança e Adolescente (NECA). Divinópolis, MG, Brasil

**Keywords:** Hope, Chronic Disease, Child, Caregivers, Nursing, Qualitative Research, Esperanza, Enfermedad Crónica, Niño, Cuidadores, Enfermería, Investigación Cualitativa, Esperança, Doença Crônica, Criança, Cuidadores, Enfermagem, Pesquisa Qualitativa

## Abstract

**Objective:**

to identify the factors that promote and threaten Hope in family caregivers of 2- to 3-year-old children with chronic conditions.

**Method:**

qualitative study with 46 family caregivers of children between 2 and 3 years old with a chronic condition, discharged from two Neonatal Intensive Care Units. Data was collected through semi-structured interviews guided by the Model for Intervention in Mutual Help Promoter of Hope. Data were submitted to deductive thematic analysis.

**Results:**

the following were identified as factors that promote Hope: The experience shared with members of the social support network; The relationship with the child; Clinical improvement of the child; Spirituality; Positive guidance for the future. The following were identified as factors that threaten Hope: Conflictual relationships and discredit of the child by close people; Uncertainties about the future; Insecurities about the ability to care for the child.

**Conclusion:**

the threatening factors of Hope generated suffering, pain, anguish, anxiety, and loneliness in caregivers. The promoting factors of Hope generated comfort, motivation, strength and joy. The findings allow Nurses to recognize the strengths and weaknesses of caregivers and adopt behaviors that promote Hope in caregivers of children with chronic conditions.

## Abstract (es)

**Objetivo:**

identificar los factores que promueven y amenazan la Esperanza en cuidadores familiares de niños de 2 a 3 años con condiciones crónicas.

**Método:**

estudio cualitativo, incluyendo 46 cuidadores familiares de niños con condición crónica de 2 a 3 años, egresados de dos Unidades de Cuidados Intensivos Neonatales. La recolección de datos ocurrió mediante entrevistas semiestructuradas guiadas por el Modelo de Intervención en Ayuda Mutua Promotora de Esperanza. Los datos fueron sometidos a análisis temático deductivo.

**Resultados:**

se identificaron como factores promotores de la Esperanza: la experiencia compartida con los miembros de la red social de apoyo; la relación con el niño; mejoría clínica del niño; espiritualidad; orientación positiva para el futuro. Se identificaron como factores amenazadores de la Esperanza: relaciones conflictivas y incredulidad con relación al niño por parte de personas cercanas; incertidumbres sobre el futuro; inseguridades sobre la capacidad de cuidar al niño.

**Conclusión:**

los factores amenazadores de Esperanza generaron sufrimiento, dolor, angustia, ansiedad y soledad en los cuidadores. Los factores promotores de Esperanza generaron consuelo, motivación, fuerza y alegría. Los hallazgos permiten a los enfermeros reconocer las fortalezas y debilidades de los cuidadores y promover la adopción de comportamientos que promuevan la Esperanza en los cuidadores de niños con condiciones crónicas.

## Abstract (pt)

**Objetivo:**

identificar quais são os fatores promotores e ameaçadores da Esperança em cuidadores familiares de crianças de 2 a 3 anos com condições crônicas.

**Método:**

estudo qualitativo, incluindo 46 cuidadores familiares de crianças com condição crônica de 2 a 3 anos egressas de duas Unidades de Terapia Intensiva Neonatal. A coleta de dados se deu por meio de entrevista semiestruturada orientada pelo Modelo de Intervenção em Ajuda Mútua Promotor de Esperança. Os dados foram submetidos à análise temática dedutiva.

**Resultados:**

foram identificados como fatores promotores da Esperança: A experiência compartilhada com membros da rede de apoio social; A relação com a criança; Melhora clínica da criança; Espiritualidade; Orientação positiva para o futuro. Foram identificados como fatores ameaçadores da Esperança: Relações conflituosas e descrença da criança por pessoas próximas; Incertezas sobre o futuro; Inseguranças sobre a capacidade de prestar os cuidados à criança.

**Conclusão:**

os fatores ameaçadores da Esperança geraram sofrimento, dor, angústia, ansiedade e solidão nos cuidadores. Os fatores promotores da Esperança geraram conforto, motivação, força e alegria. Os achados possibilitam que Enfermeiros reconheçam as potencialidades e fragilidades dos cuidadores e promovam a adoção de comportamentos promotores de Esperança em cuidadores de crianças com condições crônicas.

**Highlights:**

**(1)** Hope strengthens families of children with chronic conditions.

**(2)** There are factors that can promote or threaten parental Hope.

**(3)** The promoting factors of Hope generate comfort, motivation, strength and joy.

**(4)** The threatening factors of Hope generate suffering, anguish, anxiety and loneliness.

**(5)** Nurses can promote Hope and help families.

## Introduction

The increasing use of new health technologies and the growing specialization of maternal and child care have increased the survival of premature infants with congenital malformations and perinatal conditions ^( 1)^ . Adverse conditions in the beginning of life contribute to the development of morbidities and chronic conditions ^( 1)^ . In this study, any biological, psychological or cognitive alteration that leads to disability and residual deficits that last or have the potential to last at least one year is considered a chronic condition ^( 2, 3)^ . 

It is known that children with chronic conditions (CCC) need more care than children of the same age, as they require continuous medications, technological devices and specialized care^( 4)^. In this context, the family members are responsible for ensuring the comprehensiveness and continuity of care, meeting the demands of the child, especially in the household ^( 4, 5)^ . Thus, care for CCC generates duties for the family and requires redefinition of roles, availability of time, financial reorganization and new routines from all family members^( 6)^, but especially from the one with the main responsibility of caring, who is called main caregiver or family caregiver ^( 5)^ . 

Hope is a dimension of spirituality that is essential for successfully dealing with illness and is an active part of the anticipation, desire, and expectation of a possible positive future ^( 7)^ . In this study, Hope is defined as a multidimensional dynamic vital force that is central to life, highly personalized and future-oriented, which provides empowerment and is associated with external help, care and faith ^( 8)^ . As it is an experience of meaning for life, Hope is essential in the process of health and illness^( 9)^. It is a resource for comfort that contributes to coping with crisis and alleviates the cycle of suffering^( 10)^. 

Thus, it is understood that the factors that promote Hope are those that trigger feelings of well-being, confidence and encouragement and help in the recognition of the person’s own inner potential and in the attribution of value to life ^( 11)^ . The factors that threaten Hope are those that reduce/jeopardize Hope, generating suffering, pain, spiritual anguish, fatigue, anxiety, social isolation and loneliness ^( 11)^ . 

Hope as a dimension of and for care has gained increasing attention in Nursing, despite it being a concept already used since the 1980s ^( 7)^ ^( 9)^ . Diagnoses associated with Hope have been included in the main nursing classifications and taxonomies ^( 12)^ ^( 13)^ ^( 14)^ ^( 15)^ , but there are still few studies addressing the experience of Hope among parents of CCC. For example, authors focus on Hope as an essential and protective factor for mental health in the context of end-of- life care of CCC ^( 10)^ (16) ^( 17)^ . In Portugal, mutual-help groups for parents of CCC had influence in promoting and threatening Hope, highlighting that the support of the social network, spirituality and the ability to recognize life possibilities can promote hope, while uncertainty about the future and about the very ability to care for the child can threaten it ^( 11)^ . In Brazil, a few studies explore factors that promote Hope in caregivers of CCC ^( 18)^ ^( 19)^ , but none address the factors that threaten it. 

This investigation is based on the assumption that actions aimed at promoting Hope contribute to the care of CCC, considering the challenges imposed by the management of the chronic condition in childhood ^( 18)^ . It is understood that when health professionals do not incorporate the concept of Hope in their practices, the care offered to children and their families may be compromised. Thus, the possibilities of identifying factors that promote and threaten Hope among family caregivers of CCC are reduced; the identification of these factors is fundamental to develop individualized interventions that strengthen individuals’ internal and external coping resources and, consequently, reduce human suffering ^( 16)^ ^( 19)^ ^( 20)^ . 

Given the existing gap in scientific literature on factors that promote or threaten parent’s Hope, and considering Hope as a protective factor for mental health, which can support caregivers of CCC, the relevance of this study is justified. That said, the research question was: What are the factors that promote and threaten the Hope of family caregivers of CCC? To answer this question, the study aims to identify the factors that promote and threaten Hope in family caregivers of 2- to 3-year-old children with chronic conditions.

## Method

### Study design

Qualitative, descriptive, exploratory study, following the recommendations of the Consolidated Criteria for Reporting Qualitative Research (COREQ) ^( 21)^ . 

### Participants

The study included 46 family caregivers of CCC between 2 and 3 years old, who were discharged from two Neonatal Intensive Care Units (NICU) in Belo Horizonte, in a federal hospital and in a philanthropic hospital, selected for being references in the care of women, children and infants in the state of Minas Gerais.

### Inclusion criteria

The inclusion criteria were: being the child’s family caregiver, residing in the same household as the child, being over 18 years old. The family caregiver was the family member who had the main responsibility for the daily care of the child ^( 5)^ . The exclusion criteria were having a deficit in communication and psychological and/or psychiatric alterations that compromised the understanding of the investigative procedures. 

### Data collection

The caregivers of CCC were identified through the analysis of all the medical records of children who were discharged from the NICU from December 2016 to December 2017. This period was determined to enable the identification of children aged between 2 and 3 years. The age group was defined considering that changes in neurological, psychological and motor development are more noticeable in this age group ^( 22)^ . Subsequently, from October 2019 to May 2020, the caregivers of all identified children were contacted by telephone and the Questionnaire for Identifying Children with Chronic Conditions - Revised (QuICCC - R) ^( 23)^ was applied to confirm the chronic condition. 

A total of 1115 caregivers were identified through the analysis of medical records. Of these, 829 could not be reached, as the telephone number registered did not exist or no longer belonged to the family member. A total of 286 caregivers were contacted. It was found that 5 children had died, 218 did not have chronic conditions according to the QuICCC-R, and 63 met the conditions established by the QuICCC -R ^( 23)^ . Of these 63 children, 8 caregivers refused to participate and 9 did not meet the inclusion criteria. Thus, 46 family caregivers of CCC participated in the study. 

### Study setting

The participants were interviewed individually in a place of their choice, which was usually the family’s home, where five interviews were carried out, or a previously scheduled telephone contact, with 41 interviews in this modality. The recruitment of caregivers and the interviews were carried out by a researcher with experience in this technique and in the theme of care of CCC, who had no prior link with the participants or with the institutions where the medical records were collected.

### Instruments used for data collection

Data was collected through the application of a questionnaire for socio-demographic characterization of the families, followed by a semi-structured interview based on the Model for Intervention in Mutual Help Promoter of Hope (MIAMPE) ^( 11)^ . The script has six questions, described in Figure 1. 

**Figure 1 -:** Semi-structured interview script. Belo Horizonte, MG, Brazil

1) Tell me a story about a special moment when you felt Hope when dealing with your child’s illness.
2) What factors do you consider responsible for the increase of Hope when you feel faith, optimism and inner strength?
3) What factors do you consider responsible for the decrease of your Hope?
4) Currently, is there anyone or anything that gives you Hope when caring for your child?
5) Currently, is there anyone or anything that threatens your Hope when caring for your child?
6) How have your past experiences of Hope (when you felt full of inner strength) helped to cope with your child’s illness?

Source: Adapted from MIAMPE ^( 11)^

### Data processing and analysis

The interviews lasted an average of 49 minutes, ranging from 30 minutes to 1 hour and 43 minutes. They were audio-recorded and later transcribed. The text was reviewed for accuracy, comparing it with the audio. The anonymity of the participants was ensured by replacing their names with alphanumeric codes using the letters M, F, G and C to indicate mother, father, great-aunt and child, respectively, followed by the number corresponding to the order of the interview (e.g.: M4, F3, G1, C20).

Data were submitted to deductive thematic analysis^( 24)^. MaxQDA software version 2020 ^( 25)^ was used for data management and storage. The codes initially used were those recommended in the MIAMPE ^( 11)^ , a model based on the Hope experiences of parents of CCC, which points out factors that promote and threaten Hope as intervention resources in the field of Nursing. These codes were applied simultaneously and independently by two researchers to code the interviews. Discrepancies in coding were resolved and discussed with a third researcher. After coding 10 interviews and verifying the agreement between the researchers (Kappa = 0.83), the other codings were performed by one researcher. The fragments used to compose each of the themes were analyzed to verify the internal homogeneity and heterogeneity between the themes. Finally, two themes and 9 subthemes were identified. 

### Ethical aspects

The research complied with resolution 466/2012 of the Brazilian National Health Council (CNS) ^( 26)^ and was approved by the Research Ethics Committee under opinion CAAE: 12288919.0.0000.5149. Participants who were interviewed in person read the Informed Consent Form (TCLE) and then signed it in two copies, one offered to the participant and the other filed by the researcher. In the telephone interviews, the TCLE was read and the consent to participate in the research was recorded and filed in secure media, as provided for in Resolutions 466/12 ^( 26)^ and 510/2016 ^( 27)^ of the CNS, which allows oral consent and its recording. 

## Results

### Characterization of participants

A total of 42 (91.3%) mothers were interviewed, followed by 3 fathers (6.52%) and one aunt (2.18%). The mean age of the interviewees was 34 years, with a minimum of 19 and maximum of 54 years. The mean age of the children was 2 years and 8 months. The mean gestational age at birth was 34 weeks, with a minimum of 24 and a maximum of 41 weeks. The majority (67.39%) of the children were male. Most participants were married (52.17%), self-identified as “ pardos” (63.04%), had a religion (89.13%), had a family income of up to 2 minimum wages (67.39%), lived outside the capital of Minas Gerais (50%), and had a level of education of 9 years or more (86.95%). 

Of the total number of participants, 54.34% reported they were currently not working, and 60% of those were women, mothers, who were homemakers. Among the mothers who participated, 11 (23.91%) reported having other children with some condition, and 2 (4.34%) confirmed a history of death of a child younger than 5 years. Fifteen (32.6%) caregivers reported they had been informed that their child would have delayed growth and neuropsychomotor development, 20 (43.47%) reported that their children used daily medication, and 4 (8.68%) had children who needed technological devices at home.

### Promoting and threatening factors of Hope

**Figure 2 -:**
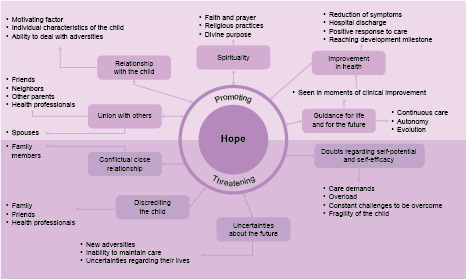
Factors that threaten or promote Hope among caregivers of Children with Chronic Conditions (CCC). Belo Horizonte, MG, Brazil

Data analysis was oriented towards the identification of factors that promote and threaten Hope, which formed the two thematic categories. The first had as sub-themes Union with others, Relationship with the child, Spirituality, Improvement in health, and Guidance for life and for the future. The category Threatening factors of Hope had as sub-themes Conflictual close relationships, Disbelief in the child, Uncertainties about the future and Doubts in self-potential and self- efficacy. A schematic overview of the themes and their respective sub-themes is shown in Figure 2. 

### Factors that promote Hope

It was found that family caregivers with other children with high-risk conditions reported a greater number of factors that promote Hope compared to those who did not have other children with high-risk conditions. Similarly, caregivers who had experienced the death of children under 5 years of age, who were religious, had a family income of more than 4 minimum wages, or had no partner identified more factors that promote Hope in the care of the CCC. The data analysis revealed information corresponding to the five factors that promote Hope in care, presented in Figure 3. 

**Figure 3 -:** Factors that Promote Hope: Description of empirical findings. Belo Horizonte, MG, Brazil

**Theme: Factors that Promote Hope**	
**Subtheme and Definition**	**Identification of empirical data**
**Union with others:** shared experience with other people	**Family members as a source of practical and emotional support** Conversations, support from family members and loved ones, prayers, that’s what gives me strength every day (M37). **Spouses as a source of practical and emotional support** My husband was my friend, my partner. He bathes her, changes diapers, gives her milk, gives her her diet when she needs it. He does everything! (M2). **Friends as a source of support** I have friends who support me a lot. In my workplace too, my co-workers always collaborate (M15). Experience shared with other parents of CCC * Each father and each mother we meet. Each story gives us strength to keep going (M13). **Health professionals who guide and follow the child** The guidelines provided by health professionals give Hope, they always explain everything in detail (M46).
**Relationship with the child:** caregivers recognize the child as a source of Hope	**The child motivates the family** My daughter is the one who always gives me Hope, joy and love (M11). **Individual characteristics of the child such as joy and excitement** Even though she has her condition, she is a happy child, she wants to jump, she wants to play, and that gives me a lot of Hope (F41). **Child’s ability to cope with adversity** In fact, he is a life lesson, we say he is a warrior. Hope is in being with him 24 hours a day (M36).
**Spirituality:** spiritual and religious beliefs and practices	**Faith and prayer as a source of strength and optimism** What supported me was faith in God. If you have faith, you have Hope, if you have Hope, you have strength, you have to believe and be optimistic (M5). **Religious practices in search of support and answers to questions** I have a lot of faith in Our Lady of Aparecida. Whenever I have a problem, I turn to her (G27). **Divine purpose** I have hope that God has a purpose for her here. That’s why he is doing miracles, healing her little by little (M17).
**Improvement in health:** the evolution and development of the child, a treatment that improves their quality of life, as well as hospital discharge	**Hospital discharge** I felt Hope when I took him out of the NICU † (M19). **Child’s positive response to health care received** I had hope the day they took his appliances off, you know? I thought: God is giving an answer. He will survive (M9). **Reaching developmental milestones and perceiving the child’s progress** I had hope when he started talking, when he took the first step (M5).
**Guidance for life and for the future:** recognizing the possibilities in life, finding paths that lead to desired goals, and being motivated to follow these paths	**Continuing care** The hope I have is that she will walk, you know? So I take her to physical therapy and I always encourage her, stimulate her (M16). **Acquisition of autonomy by the child** I have hope that they will have a life as close to normal as possible, that they will be independent, that they will be able to work (M15). **Development of children** The fact that he knows us, he understands what we say, he wants to talk. These things give me hope, make me believe in his improvement, you know? (M4).

* CCC = Children with Chronic Conditions

† NICU = Neonatal Intensive Care Unit

### Union with others

It consists of experiences shared with other people, from the family or from their social network, which may include friends, health professionals and other parents of CCC. Participants report that the family is a solid and helpful support network, which gives strength (M20), offers support (M28), is solidary (F41) and is always available when needed (M37). Within the family, spouses stand out as a source of emotional and practical support, as they help with care (M15) , fight alongside (M2), and are always sharing the laughs and experiences, the moments of pain and joy (M22). The children’s grandmothers were also identified as a source of Hope, for providing help (M15) and support (M15), as they stay with my son when I need it (M1), always keep me company (M18) and tell me that everything will be fine, that my son will get better (M38). 

The children’s uncles and cousins were also identified as promoters of Hope. M2 states that uncles and cousins are always available to help (M2), M10 says that she received help (M10) from her brother after childbirth and M17 reported that her cousin takes care of C17 with great dedication and affection (M17). Similarly, friends, co-workers, and people connected to religious institutions are also very supportive (M15), help in prayer (M9) and hug me as if I were family (M11). 

Health professionals are also referred to as people who promote Hope. This because they follow the evolution (M5) of the child, share the same goals (M6), are very competent and experienced (M7), provide calmness (G27) in the family, and are very positive (M28). Furthermore, experiences shared with family members of other children who live in similar situations were a source of support, and each father and mother we meet gives us the strength to keep going (M13). 

### Relationship with the child

Family members recognize the CCC themselves as source of Hope, as they bring Hope that everything will be fine (M37), give a lot of strength (M14), generates comfort (M14) and renews faith (M26). In addition, caregivers indicate the characteristics of children that favor the feeling of Hope, such as joy and motivation (M11) and daily smiles (F41). The strength they convey in their daily lives and their will to live make caregivers refer to them as sources of Hope: she is a warrior, a very energetic and smiley girl (M21), he has enormous willpower. I’ve never met a child with so much determination (M16). 

### Improvement in health

Moments of clinical improvement in the child’s health and hospital discharge are identified as events that promote Hope. Thus, the child’s survival, after the possibility of losing their life, leads the family to believe in progressive improvement, as evidenced by M14: I’ll have hope for my whole life that she will see. When she was born, they told me there was a zero percent chance she would survive, and now she’s here bursting with health! (M14). Caregivers also report that their Hope was enhanced when the child reached developmental milestones, such as talking and walking (M5), moving their arms (M13), and answering when their parents call (M18), and that seeing the child’s development helps to endure another day (M1). 

Improvements in the child’s quality of life, attained with the care received, are also seen as promoters of Hope. It is shown that either the use of new technologies or the suspension of their use promotes Hope, if they contribute to improving the child’s quality of life. M9 and M10 mentioned the suspension of CPAP (Continuous Positive Airway Pressure) and oxygen, and M7 mentioned the reduction of medication. Hospital discharge was seen as contribution for the promotion of Hope, which was evidenced when the participants mentioned that this was a moment of celebration and joy (M1) and a sign that the child is fine (M19). 

### Spirituality

Spiritual and religious beliefs and practices such as faith, prayer and participation in religious activities were identified as factors that promote Hope. The interviewees mentioned that believing in a divine being was a factor that increased Hope, strength (M37) and optimism (M5). Through the exercise of faith, families resort to a superior being, who is referred to as a foundation for Hope (M11), a source of help (M24) and the one who is in control of everything (M24) and helps them to persist and not give up (M11). It is evident that this belief leads caregivers to also believe in the cure of their children’s health condition, based on their own experiences or experiences of other people, God healed the crippled, healed the blind, why wouldn’t He heal him? (M4), for God, nothing is impossible (F40). 

### Guidance for life and for the future

Guidance for life and for the future consists of establishing or reformulating goals and objectives. It is the perceived ability to recognize possibilities in life to achieve your goals. As a way of recognizing such possibilities, the participants indicate what they envision for their children in the future, such as Talking (M10), Walking (M42), Eating (M17), Seeing (M14), Having a normal life and being independent (M15) and No longer needing so much help (M25). Monitoring and stimulation of the child’s development appear as routes to achieve these goals: She undergoes physiotherapy, and as a mother, I follow this evolution, which gives more strength to seek her treatment and to believe that she will walk one day (M20). 

### Factors that threaten Hope

It is observed that family caregivers who had no other children with high-risk conditions mentioned factors that threaten Hope more frequently than participants who already had children in this condition. A similar trend was found in the speeches of caregivers whose children had delayed growth and development, used technological devices, or were on continuous medication. The information of the subtopics is shown below in Figure 4. 

**Figure 4 -:** Factors that threaten hope: description of the empirical findings. Belo Horizonte, MG, Brazil

**Factors that Threaten Hope**	
**Subtopic and Definition**	**Identification of empirical data**
**Conflictual close relationships:** relationships that generate negative feelings such as pain, anguish, loneliness, anxiety and sadness	**Spouses in extramarital relationships** He got involved with other women when I was in the hospital with my son. He threatened my feelings, I don’t know if that can be healed (M1). **Derogatory remarks about the child from family members** My mother-in-law and my sister-in-law treat my son as if he were incapable; they say things that hurt me a lot (M16).
**Discrediting the child:** third partied discrediting the child’s potential or the evolution of their clinical condition	**Family members** My mother says he will be debilitated. She has this pessimistic view. She says he will never play soccer (M7). **Friends** I feel this from people around me. It’s a kind of prejudice regarding my son’s use of hearing aids (M13). **Health professionals who discredited the survival and development of the children** They told us that she wouldn’t walk, she wouldn’t talk (F33).
**Uncertainties about the future:**undesirable events that can compromise the child’s health condition and the families’ condition to maintain care	**New adversities related to child health** For me, the only threat is the disease, I’m afraid it will keep progressing, even with the treatment (M45). **Uncertainties about their lives** Fear! (Mother cries). I’m very afraid of losing her, I’m afraid she won’t live long (M24).
**Doubts regarding self-potential and self- efficacy:**family’s insecurities and perceptions about inability to help and lack of confidence to provide care	**Required care** He was so tiny to need oxygen. He even had to come home with the oxygen. And it was extremely difficult for me (M10). **Fragility of the child** I was afraid because he was very small and I couldn’t take care of him. I was like: How to take care of something so little? (M1). **Overload related to care demands** Sometimes we are so overloaded with work, you know? There are many demands. Sometimes you get really discouraged (M15). **Inability to maintain care due to financial difficulties** Unemployment complicates everything, there are things she needs that I cannot buy (F41).

### Uncertainties about the future

These are undesirable events that can compromise the children’s health and the families’ condition to maintain care. It is observed that caregivers are concerned with the possibility of the children’s health getting worse as a result of disease progression or less response to treatment is evident. In this topic, M45 considers that the only threat is the disease, I’m afraid it will keep progressing, even with the treatment (M45). M38 fears that he will run out of the medication and get worse (M38). Similarly, G27 is afraid he may need hemodialysis (T27). 

The participants also feared the children’s death, which made it even more difficult for them to Hope for a better future. This can be seen in the following speeches: I’m very afraid of losing her, I’m afraid she won’t live long (M24), I am very afraid of losing him. So I can’t see a ray of Hope yet (M31). 

### Conflictual close relationships

They are referred to as people close to the caregiver who threaten their Hope through attitudes that generate pain, anguish, loneliness, anxiety, and sadness. The participants mentioned conflicts with their spouse due to his involvement with other women, which has brought up a lot of bad feelings (M1) and aggressiveness: he is very aggressive with me (M19). 

Other family members were also identified as threats to Hope. In general, this threat is related to derogatory remarks or disbelief in the child’s improvement: Whenever I bring her news about C16, my mother-in-law says: Let’s see where it goes. She is a person who does not see what he can become. She thinks he’s debilitated. She takes away my Hope. Both her and her daughter (M16). In addition, people who do not contribute to care or do not respect the child’s recommended diet were considered threats to Hope: People have no respect. If you offer whole milk to C18, you are putting his life at risk, he gets diarrhea for 15 days, and people do not respect this and offer it (M18). 

### Disbelief in the child

It refers to parents’ awareness of other people disbelief in the potential and the improvement of their children. This occurred even before the child was born: The doctor said he would have only one hour to live (M16), The doctor told me that if she were to live, she would not be able to walk, she would not be able to speak, she would not be able to breathe on her own. She could only live with life support. So I asked: Does that mean it’s better not to survive at all? and the doctor said: It’s better! (M14). 

Professionals also disbelieved in the child in outpatient follow-ups, stating that they would not be able to move any of their limbs (M8), would not walk (F33) and would not speak (P33). The participants also perceive that family members and other close people disbelief in the child’s evolution. M7’s mother says that the child will be debilitated (M7) and will not be able to engage in activities such as playing (M7) and M13 says that there is prejudice regarding my son’s use of hearing aids (M13) among her friends. 

### Doubts regarding self-potential and self-efficacy

Insecurities about their internal resources and their ability to develop new skills to care for their children, as well as the lack of confidence to perform care, represent threats to Hope. The fear of taking care of the child was related to the fragility attributed to the child, who was identified as very small (M11) and fragile (M26). Participants expressed doubts about their ability to care, asking themselves: How am I going to take care of this girl at home? (M26), How will I learn to take care of him? (M1). 

Finally, unemployment and lack of money to cover the child’s expenses are mentioned as factors that reduce their Hope for the future. This situation can be observed in the speech of F41: Unemployment makes me a little upset, because everything is an expense with her, and if you are unemployed you are limited (F41). 

## Discussion

This study allowed identifying factors that promote and threaten Hope in caregivers of CCC. In addition, it confirmed the assumption that Hope is an essential dimension of care, enhanced in times of crisis, such as in the context of a chronic condition in childhood. Therefore, Hope appears in the path of care of CCC and is seen by family caregivers as a factor that helps maintain a positive perspective and that can be stimulated or compromised considering the context and the encounters.

In its investigation of Hope, the present study found that family caregivers were predominantly female. Other studies also indicate that the burden of chronic disease constantly falls on the mother, who is usually the main caregiver ^( 5)^ ^( 28)^ . In addition, in this study, most mothers dedicated themselves exclusively to home and child care, which is in line with findings in the literature that indicate that women stop working to stay at home due to the demands of the child ^( 5)^ ^( 28)^ . 

Caregivers indicate that they need external support to promote or maintain Hope, sharing difficulties and receiving emotional and practical support, as pointed out in studies that explored Hope in parents of children with cancer ^( 29, 30)^ . Thus, the support received from family members, spouses, friends ^( 29– 31)^ and health professionals ^( 30)^ is important to provide encouragement and comfort ^( 30)^ . On the other hand, the lack of a social support network reduces the Hope of these caregivers. A qualitative study that investigated the experience of mothers when caring for children with leukemia, concluded that the lack of social support causes suffering for caregivers and compromises their ability to face challenges ^( 32)^ . 

Support groups with other parents of CCC are also cited in the literature as important resources for promoting Hope ^( 11, 29)^ . Engaging with other caregivers with similar experiences encourages the sharing of ideas, information and concerns^( 11)^. In addition, the exchange of experiences with other parents of CCC lessens the feeling of social isolation and increases the feeling of identification, helping caregivers to have a more positive perspective on the child’s illness and to envision new possibilities ^( 32)^ . 

The results of this study also demonstrate that health professionals can help promote or reduce the Hope of these parents. These professionals help to promote hope through the empathic sharing of truthful, understandable and objective information, while lack of empathy in communication and omission of information have the potential to reduce the family’s Hope ^( 31)^ . 

The dimension of spirituality, namely faith and trust in God, is considered by the participants as crucial for maintaining Hope. Thus, families nourish their Hope through religious practices ^( 30, 32)^ . The belief that the experience of the chronic illness has a divine purpose and that the child’s future can be promising helps caregivers to maintain Hope ^( 31)^ . 

The reports of the caregivers also refer to Hope as a view towards a future based on goals, which are sometimes related to expectations regarding the improvement of the disease or the survival of the child. This way, Hope works as an anticipation of a future with better possibilities than the past and the present^( 34)^. For the participants of this study, the ideal future would bring a sense of normality. Findings indicate that parents’ Hope is, in general, related to curing the child, with the possibility of stabilizing the disease, achieving better quality of life and normality, and building a better future without suffering ^( 17)^ ^( 29)^ . 

The caregivers of CCC often report feelings of uncertainty, which appear as an unexpected future ^( 35)^ . A study carried out with parents of children in palliative care pointed out that the family’s experience of Hope was filled with uncertainties, especially regarding concerns with worsening of the health condition and death, which lead to an abrupt loss of Hope ^( 36)^. Furthermore, caregivers also feel insecure about their own ability to manage and provide care, as they do not believe they have the potential to carry out care measures successfully. Therefore, learning about this care and having support from health professionals during the child’s hospitalization are essential for caregivers to develop and strengthen their skills^( 36)^. 

The children were also identified as a source of Hope by their caregivers. This is because the way the child deals with the chronic condition works as a coping model for the caregiver^( 37)^. Finally, this study concluded that caregivers who had had other children with high-risk conditions or a history of death of children under 5 years of age cited more factors that promote Hope than those who had not. Thus, Hope appears in studies as a resilience factor and a strategy for coping with and adapting to stressful situations, such as caring for a CCC. A study carried out with parents of children with cancer found that when parents come to terms with the diagnosis of their children they develop a resilient mindset that facilitates the process of coping with chronic illness in childhood^( 38)^. On the other hand, those who did not adapt to the diagnosis had a decrease in Hope and in the ability to deal with the disease^( 38)^.

As a contribution, the present study can help nurses to identify the strengths and weaknesses of caregivers of CCC and support them in the daily care of these children. Therefore, recognizing the importance of Hope in the health-disease process can contribute to the implementation of actions that promote Hope and more effective interventions aimed at the adaptation of families to the challenges of chronic illness.

Therefore, considering the factors that promote and threaten Hope revealed in this study, Nursing professionals should encourage and support the participation of families of CCC in mutual support groups, assist in the identification of their support network, reinforce the positive advances in the child’s development, provide clear and objective information about the child’s condition, help in the construction of plans for the future, develop qualified listening to identify the caregivers’ needs, prepare the caregiver for the management of care at home and, when necessary, activate the health care network for better monitoring and support for the family.

A limitation of this study is the non-participation of other family members, who could provide different points of view. Future studies should include other family members, health professionals, and children with different health conditions, to allow investigating common and differential aspects related to Hope. In addition, longitudinal studies should be carried out to investigate the feeling of Hope in the different stages of the care trajectory of CCC.

## Conclusion

Caregivers of CCC perceive Hope throughout the child’s care path and claim that there are strategies capable of promoting or threatening that Hope. The promoting factors generate comfort, motivation, strength and joy for caregivers. Threatening factors, on the other hand, generate suffering, pain, anguish, anxiety and loneliness. Therefore, care measures aimed at promoting Hope have an important role in this context, considering the nature of the chronic disease, the limitations and challenges related to the daily management of symptoms, the therapeutic regimen, and the care demands.
